# Risk factors and outcomes of cardiovascular disease readmission within the first year after dialysis in peritoneal dialysis patients

**DOI:** 10.1080/0886022X.2020.1866009

**Published:** 2021-01-13

**Authors:** Jianbo Li, Naya Huang, Zhong Zhong, Pema Joe, Dan Wang, Zhen Ai, Lisha Wu, Lanping Jiang, Fengxian Huang

**Affiliations:** a Department of Nephrology, The First Affiliated Hospital, Sun Yat-sen University, Guangzhou, China; b Key Laboratory of Nephrology, National Health Commission and Guangdong Province, Guangzhou, China; c Department of Medicine, Linzhi People’s Hospital, Linzhi, China

**Keywords:** Cardiovascular disease readmission, peritoneal dialysis, risk factors, outcomes

## Abstract

**Background:**

In the first year of dialysis, patients are vulnerable to cardiovascular disease (CVD) hospitalization, but knowledge regarding the risk factors and long-term outcomes of cardiovascular readmission within the first year after dialysis in incident continuous ambulatory peritoneal dialysis (CAPD) patients is limited.

**Methods:**

This retrospective cohort study was conducted in incident CAPD patients. The demographic characteristics, laboratory parameters, and CVD readmission were collected and analyzed. The primary outcome was all-cause mortality, and the secondary outcomes included CVD mortality, infection-related mortality and technique failure. A logistic regression was used to identify the risk factors associated with CVD readmission within the first year after dialysis. Cox proportional hazards models were used to evaluate the association between CVD readmission and the outcomes.

**Results:**

In total, 1589 peritoneal dialysis (PD) patients were included in this study, of whom 120 (7.6%) patients had at least one episode of CVD readmission within the first year after dialysis initiation. Advanced age, CVD history, and a lower level of serum albumin were independently associated with CVD readmission. CVD readmission within the first year after dialysis was significantly associated with all-cause (HR 2.66, 95%CI 1.91–3.70, *p* < 0.001) and CVD (HR 3.42, 95%CI 2.20–5.31, *p* < 0.001) mortality, but not infection-related mortality or technique failure, after adjusting for confounders.

**Conclusions:**

Our findings suggest that an advanced age, a history of CVD, and a lower level of serum albumin were independently associated with CVD readmission. Moreover, CVD readmission was associated with all-cause and cardiovascular mortality in incident CAPD patients.

## Introduction

Dialysis patients have more comorbidities than the general population and may require more hospital care [1–3]. The United States Renal Data System (USRDS) annual report showed that patients with end-stage renal disease (ESRD) were admitted to the hospital at a rate of 1.7 per patient-year, and 27% of the annual hospitalization expenditures were due to cardiovascular conditions [4].

Peritoneal dialysis (PD) patients have been shown to have a relatively high prevalence of readmission ranging from 15.5% to 37.4% in previous studies [5–7]. Cardiovascular disease (CVD) accounts for a large proportion of readmissions in dialysis patients [4,8–10], especially within the first year after dialysis [10]. A study involving in hemodialysis (HD) patients noted that patients on dialysis for less than one year had an increased risk of pulmonary edema-related readmissions compared with those on dialysis for more than one year [10], which increases vulnerability to CVD events within the first year of dialysis. Little data explored CVD readmission within the first year in PD patients; however, it has been demonstrated that changes from ESRD to PD may confer a risk of congestive heart failure, arterial stiffness and other CVD events to PD patients initiating dialysis [11].

Readmission is a reflection of patients’ health condition and has been shown to be associated with increased morbidity and mortality and a reduced quality of life among dialysis patients [2,9,12–14]. However, some studies have shown that dialysis patients are at a high risk of readmission, but long-term outcomes may improve due to close post-discharge monitoring [8]. Moreover, a study involving patients with heart failure, acute myocardial infarction and pneumonia suggested that a reduction in readmission rates was associated with increased 30-day post-discharge mortality [15], indicating that readmission care can improve outcomes.

Although several studies focused on the burden, correlates and outcomes of readmission in PD patients [6–9,13,14], these studies mainly considered short-term readmission with durations ranging from 7 to 120 days. Knowledge regarding the incidence, risk factors and long-term outcome within the first year after dialysis in PD patients, especially CVD readmission, is limited. In this study, we focus on the incidence of CVD readmission within the first year after dialysis initiation and evaluate its risk factors and effects on long-term outcomes in PD patients.

## Materials and methods

### Study population and data sources

This was a retrospective, single-center cohort study. Patients who underwent PD catheter implantation in the Department of Nephrology, The First Affiliated Hospital of Sun Yat-sen University from 1 January 2006, to 31 December 2013, were eligible to participate in the study. Patients aged ≥18 years, on PD for more than 3 months, and who were regularly followed up in our PD center were included, and those with a history of kidney transplantation, who had undergone HD for more than 3 months, or had malignant diseases were excluded. All patients were followed up until death, withdrawal of PD, or 31 December 2019. This study was carried out in accordance with the Declaration of Helsinki, and the study protocol was approved by the Ethics Committee of The First Affiliated Hospital, Sun Yat-sen University ([2016]215). Written informed consent was obtained from all participants.

### Demographic and clinical data

The baseline demographic characteristics and laboratory data were collected within 3 months after dialysis initiation. The demographic data included sex, age, primary cause of ESRD, history of diabetes mellitus (DM), CVD, hypertension, and body mass index (BMI). CVD was defined as congestive heart failure, acute myocardial infarction, unstable angina, malignant arrhythmias, valvular heart disease, ischemic stroke, cerebral hemorrhage, transient ischemic attack and peripheral vascular disease [16,17]. The laboratory data included hemoglobin, albumin, calcium, phosphorus, intact parathyroid hormone (iPTH), total cholesterol (TC), triglyceride, high density lipoprotein cholesterol (HDLC), low density lipoprotein cholesterol (LDLC), sodium, potassium, normalized protein catabolic rate (nPCR), weekly total Kt/V urea, residual renal function (RRF) measured as residual renal creatinine clearance in ml/min/1.73 m^2^ and total weekly creatinine clearance (WCCr).

### Index admission and CVD readmission

The index admission was defined as the admission during which the patients underwent PD catheterization and started the PD treatment. Unexpected readmission within the first year was defined as readmission due to unexpected clinical events within 365 days after the index discharge. Scheduled admissions, such as reexaminations or other planned procedures, were excluded. CVD readmission was defined as readmission due to congestive heart failure, myocardial infarction, angina, arrhythmias, valvular heart disease, ischemic stroke, cerebral hemorrhage, transient ischemic attack and peripheral vascular disease [16].

### Outcomes

The long-term outcomes included all-cause mortality, CVD mortality, infection-related mortality and technical failure. CVD mortality was defined as mortality caused by CVD events and sudden death. Infection-related mortality was defined as mortality caused by peritonitis or other infections. Death-censored technical failure was defined as a switch to HD for more than 90 days due to any cause, including inadequate dialysis, ultrafiltration failure, intractable peritonitis, exit-site and/or tunnel infection, catheter malfunction, mechanical problems such as hernia or abdominal operation, etc. and it was censored for death, spontaneous recovery of renal function, move to another center, kidney transplantation, and/or ‘still on PD’ until 31 December 2019 [18].

### Statistical analysis

The baseline continuous data were expressed as the mean ± standard deviation and median (25th percentile, 75th percentile), and the categorial data are expressed as frequency (%). Pairwise deletion (Available Case Analysis) was used to address the missing data. Comparisons between two groups were performed by Student *t*-tests for continuous variables with normal distribution and by non-parametric Mann–Whitney test for continuous variables with skewed distribution. Categorical data were performed by Chi-square tests. A logistic regression was used to identify the risk factors associated with CVD readmission in the first year. Conditional backward stepwise was used in the multivariate logistic regression model. Kaplan–Meier curve and log(−log(survival)) graph analyses were performed to test the proportionality of the cox analyses, and the data satisfied the proportionality of the cox analyses. Cox proportional hazards models were used to estimate the association between CVD readmission and long-term all-cause, CVD, and infection-related mortality and technique failure.

## Results

### Baseline characteristics of the study cohort

**Figure 1.**
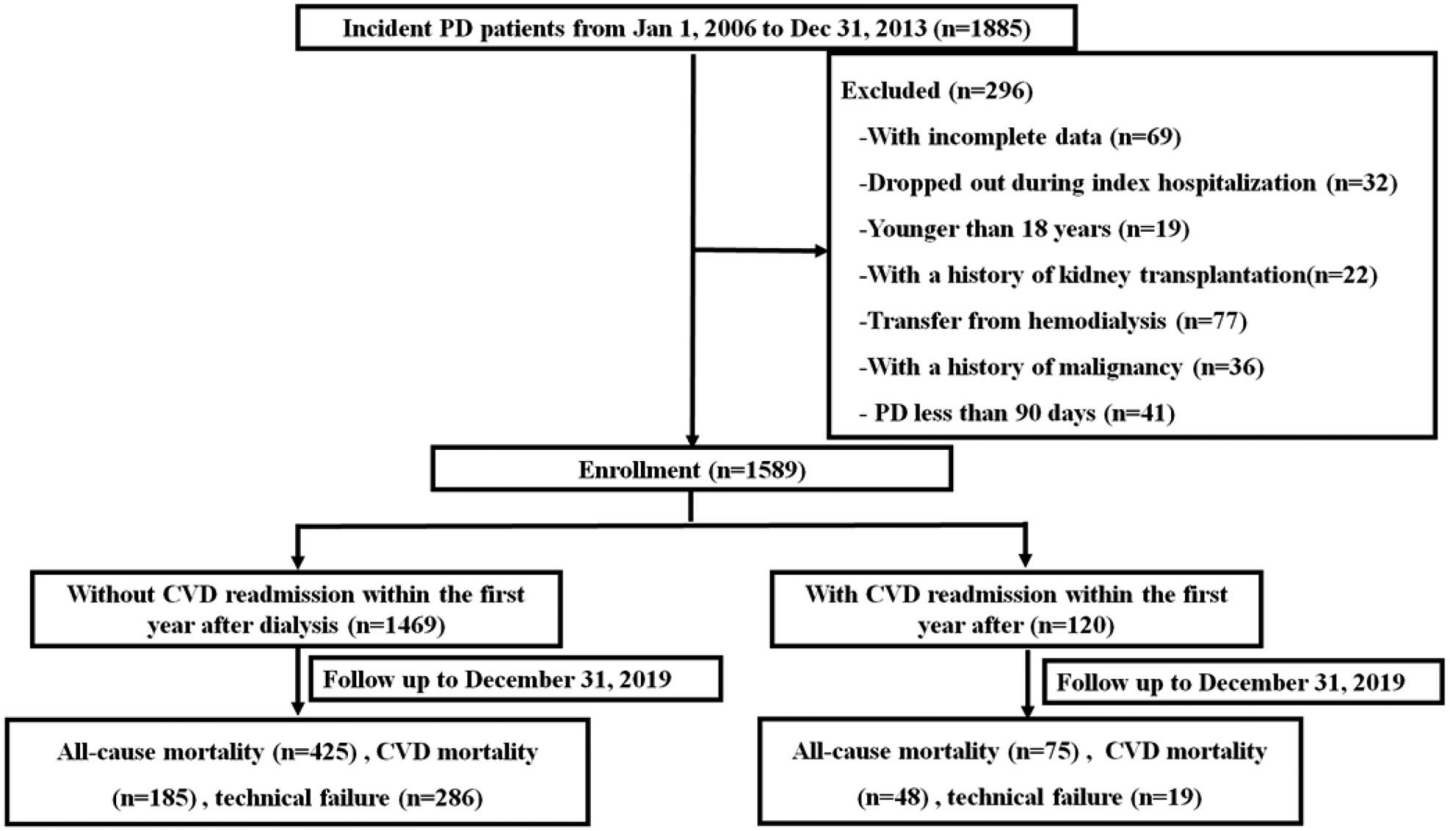
Study population selection procedures.

In total, 1885 incident CAPD patients were screened, and 1589 patients were ultimately included in this study (Figure 1). The baseline characteristics of the study cohort are shown in Table 1. Among the patients, 953 (60%) patients were male, the average age was 46.9 ± 15.3 years, 409 (25.7%) patients had a history of DM, 337 (21.2%) patients had a history of CVD, and 307 (19.3%) patients had a history of hypertension. In total, 496 (31.2%) patients had at least one episode of readmission within the first year after dialysis initiation, including 120 patients who were readmitted due to CVD. CVD readmission affected 7.6% of all patients and accounted for 24.2% of all readmissions within the first year after dialysis initiation. Compared with those without CVD readmission, the patients with CVD readmission were older (56.3 ± 15.7 vs 46.2 ± 15.0 years, *p* < 0.001), more likely to have a history of DM (42.5% vs 24.4%, *p* < 0.001), CVD (44.2% vs 19.3%, *p* < 0.001), and hypertension (35.0% vs 18.0%, *p* < 0.001), and had a higher BMI (22.5 ± 4.0 vs 21.6 ± 3.5, *p* = 0.01), but lower levels of hemoglobin (97.9 ± 17.3 vs 101.4 ± 21.5 g/L, *p* = 0.04), albumin (34.1 ± 6.0 vs 37.1 ± 5.4 g/L, *p* < 0.001), iPTH [186 (74–358) vs 252 (120–436) pg/ml, *p* = 0.003], and HDLC(1.08 ± 0.38 vs 1.22 ± 0.44 mmol/L, *p* = 0.001). However, no significant differences were observed in serum calcium, phosphorus, TC, triglyceride, LDLC, potassium, nPCR, weekly total Kt/V urea, RRF or WCCr.

**Table 1.** Baseline characteristics of the overall cohort and patients with and without CVD readmission within the first year after dialysis.

	Overall (*n* = 1589)	With CVD readmission group (*n* = 120)	Without CVD readmission group (*n* = 1469)	*p* Value
Male (n, %)	953 (60%)	78 (65%)	875 (59.6%)	0.24
Age (years)	46.9 ± 15.3	56.3 ± 15.7	46.2 ± 15.0	**<0.001**
Underlying kidney disease (n, %)				**0.001**
Glomerulonephritis	962 (60.5%)	51 (42.5%)	911 (62%)	
Diabetic nephropathy	360 (22.7%)	49 (40.8%)	311 (21.2%)	
Hypertensive nephropathy	112 (7%)	14 (11.7%)	98 (6.7%)	
Others	155 (9.8%)	6 (5%)	149 (10.1%)	
History of DM (n, %)	409 (25.7%)	51 (42.5%)	358 (24.4%)	**<0.001**
History of CVD (n, %)	337 (21.2%)	53 (44.2%)	284 (19.3%)	**<0.001**
History of hypertension (n, %)	307 (19.3%)	42 (35.0%)	265 (18.0%)	**<0.001**
BMI (kg/m^2^)	21.7 ± 3.6	22.5 ± 4.0	21.6 ± 3.5	**0.01**
Hemoglobin (g/L)	101.1 ± 21.2	97.9 ± 17.3	101.4 ± 21.5	**0.04**
Albumin (g/L)	36.9 + 5.5	34.1 ± 6.0	37.1 ± 5.4	**<0.001**
Calcium (mmol/L)	2.24 ± 0.25	2.2 ± 0.2	2.2 ± 0.3	0.16
Phosphorus (mmol/L)	1.5 ± 0.5	1.4 ± 0.5	1.5 ± 0.5	0.20
iPTH (pg/ml)	248 (119–432)	186 (74–358)	252 (120–436)	**0.003**
TC (mmol/L)	5.0 ± 1.4	4.9 ± 1.4	5.0 ± 1.4	0.58
Triglyceride (mmol/L)	1.66 ± 1.13	1.80 ± 1.22	1.64 ± 1.12	0.13
HDLC (mmol/L)	1.20 ± 0.44	1.08 ± 0.38	1.22 ± 0.44	**0.001**
LDLC (mmol/L)	2.87 ± 1.05	2.86 ± 1.18	2.87 ± 1.04	0.88
Sodium (mmol/L)	140.0 ± 3.8	139.4 ± 3.88	140.1 ± 3.78	0.05
Potassium (mmol/L)	3.8 ± 0.8	3.84 ± 0.76	3.83 ± 0.74	0.86
nPCR (g/kg/d)	1.06 ± 0.20	0.85 ± 0.21	1.08 ± 0.23	0.36
Weekly total Kt/V urea	2.45 ± 0.73	2.41 ± 0.76	2.45 ± 0.72	0.56
RRF (ml/min/1.73 m^2^)	3.2 (1.9–5.0)	2.9 (1.4–4.7)	3.3 (1.9–5.1)	0.22
WCCr (L/w/1.73 m^2^)	97.8 ± 28.2	82.5 ± 30.4	99.0 ± 29.2	0.63

The results of continuous variables are expressed as mean ± SD for those with normal distribution, median (25th percentile and 75th percentile) for those with skewed distribution, and categorical data were expressed as number (%). Comparisons between two groups were performed by Student *t*-tests for continuous variables with normal distribution and by non-parametric Mann–Whitney test for continuous variables with skewed distribution. Categorical data were performed by Chi-square tests.

CVD: cardiovascular disease; DM: diabetes mellitus; iPTH: intact parathyroid hormone; TC: total cholesterol; HDLC: high-density lipoprotein cholesterol; LDLC: low-density lipoprotein cholesterol; nPCR: normalized protein catabolic rate; RRF: residual renal function; WCCr: total weekly creatinine clearance.

Bold indicates significance at *p* < 0.05.

### Factors associated with unexpected CVD readmission within the first year after dialysis

The factors associated with unexpected CVD readmission within the first year after dialysis were analyzed and are shown in Table 2. The univariate logistic regression revealed that age [odds ratio (OR) 1.04, 95% confidence interval (CI) 1.03–1.06, *p* < 0.001], a history of DM (OR 2.29, 95%CI 1.57–3.36, *p* < 0.001), CVD (OR 3.30, 95%CI 2.25–4.84, *p* < 0.001), or hypertension (OR 2.45, 95% CI 1.63–3.64, *p* < 0.001) and BMI (OR 1.08, 95%CI 1.02–1.14, *p* = 0.009) were risk factors for unexpected CVD readmission within the first year after dialysis, while albumin (OR 0.91, 95%CI 0.88–0.94, *p* < 0.001), iPTH (OR 0.99, 95%CI 0.99–1.00, *p* = 0.014), HDLC (OR 0.48, 95%CI 0.32–0.74, *p* = 0.001) and nPCR (OR 0.17, 95%CI 0.06–0.50, *p* = 0.001) were protective factors for readmission. After the backward stepwise multivariate logistic regression, an advanced age (OR 1.03, 95% CI 1.01–1.05, *p* = 0.001), history of CVD (OR 2.18, 95%CI 1.30–3.78, *p* = 0.006), and lower level of serum albumin (OR 0.90, 95%CI 0.85–0.96, *p* < 0.001) were independently associated with CVD readmission.

**Table 2.** Factors associated with CVD readmission within the first year after dialysis.

Variables	Univariate regression		Multivariate regression	
	HR (95% CI)	*p* Value	HR (95% CI)	*p* Value
Male (vs female)	1.26 (0.85–1.86)	0.24	–	–
Age (per years)	1.04 (1.03–1.06)	**<0.001**	1.03 (1.01–1.05)	**0.001**
DM history (yes vs no)	2.29 (1.57–3.36)	**<0.001**	–	–
CVD history (yes vs no)	3.30 (2.25–4.84)	**<0.001**	2.18 (1.30–3.78)	**0.006**
Hypertension history (yes vs no)	2.45 (1.63–3.64)	**<0.001**		
BMI (per 1 kg/m^2^)	1.08 (1.02–1.14)	**0.009**	–	–
Hemoglobin (per 1 g/L)	0.99 (0.98–1.00)	0.09	–	–
Albumin (per 1 g/L)	0.91 (0.88–0.94)	**<0.001**	0.90 (0.85–0.96)	**<0.001**
Calcium (per 1 mmol/L)	0.61 (0.30–1.21)	0.16	–	–
Phosphorus (per 1 mmol/L)	0.77 (0.52–1.15)	0.20	–	–
iPTH (per 1 pg/ml)	0.99 (0.99–1.00)	**0.014**	1.00 (1.00–1.01)	0.11
TC (per 1 mmol/L)	0.96 (0.84–1.10)	0.58	–	–
Triglyceride (per 1 mmol/L)	1.11 (0.97–1.28)	0.14	–	
HDLC (per 1 mmol/L)	0.48 (0.32–0.74)	**0.001**	0.54 (0.27–1.08)	0.08
LDLC (per 1 mmol/L)	0.99 (0.83–1.18)	0.88	–	–
Sodium (per 1 mmol/L)	0.96 (0.91–1.00)	0.05	–	
Potassium (per 1 mmol/L)	1.02 (0.80–1.32)	0.86	–	
nPCR (per 1 g/kg/d)	0.17 (0.06–0.50)	**0.001**	–	
Weekly total Kt/V urea	0.92 (0.69–1.22)	0.56	–	
RRF (per 1 ml/min/1.73 m^2^)	0.93 (0.86–1.01)	0.09	–	
WCCr (per 1 L/w/1.73 m^2^)	0.99 (0.99–1.00)	0.28	0.99 (0.98–1.00)	0.05

Bold indicates significance at *p* < 0.05. A backward stepwise multivariate logistic regression was used to evaluate the associated factors. Parameters, including sex, age, DM history, CVD history, hypertension, hemoglobin, albumin, calcium, phosphorus, iPTH, TC, Triglyceride, HDLC, LDLC, nPCR, WCCr, and weekly total Kt/V urea were included in the model.

CVD: cardiovascular disease; DM: diabetes mellitus; iPTH: intact parathyroid hormone; TC: total cholesterol; HDLC: high-density lipoprotein cholesterol; LDLC: low-density lipoprotein cholesterol; nPCR: normalized protein catabolic rate; WCCr: total weekly creatinine clearance.

### Clinical outcomes in patients with and without unexpected CVD readmission within the first year after dialysis

**Figure 2.**
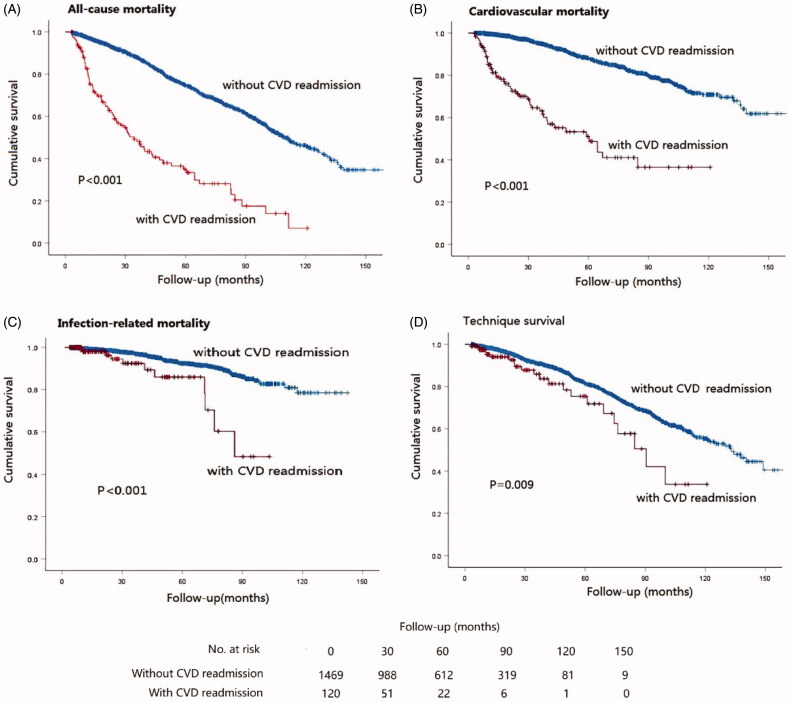
Kaplan–Meier plots of all-cause mortality, CVD mortality, infection-related mortality, technique failure and CVD readmission.

The long-term outcomes of the study cohort are shown in Table 3. During a median follow-up period of 47.2(21.2–82.2) months, 425 (29.1%) patients without CVD readmission and 75 (62.5%) patients with CVD readmission died from all causes. Of these patients, 48 (40%) patients with CVD readmission and 185 (12.6%) patients without CVD readmission died from CVD events. To evaluate cardiovascular readmissions with long-term clinical outcomes, Kaplan–Meier plots were drawn, and a cox regression was used. The Kaplan–Meier plots of unexpected CVD readmission, all-cause mortality, CVD mortality, infection-related mortality and technique failure are shown in Figure 2. The patients with unexpected CVD readmission within the first year showed poor outcomes in all-cause mortality, CVD mortality, infection-related mortality and technique failure (all *p* < 0.05 in the Kaplan–Meier plots). The Cox regression analysis (shown in Table 4) showed that cardiovascular readmission within the first year after dialysis was associated with a higher risk of all-cause mortality (HR 2.66, 95% CI 1.91–3.70, *p* < 0.001) and CVD mortality (HR 3.42, 95%CI 2.20–5.31, *p* < 0.001) after adjusting for substantial confounders, including age, sex, DM history, CVD history, hypertension history, BMI, hemoglobin, albumin, calcium, phosphorus, TC, triglyceride, HDLC, LDLC, sodium, potassium, WCCr and RRF. Moreover, cardiovascular readmission within the first year after dialysis was associated with a higher risk of infection-related mortality and technique failure after adjusting for age and sex (HR 2.05, 95%CI 1.08–3.90, *p* = 0.03; HR 1.60, 95%CI 1.00–2.55, *p* = 0.05, respectively), but the significance was attenuated after adjusting for DM history, CVD history, hypertension history, BMI, hemoglobin, albumin, calcium, phosphorus, TC, triglyceride, HDLC, LDLC, sodium, potassium, WCCr and RRF (HR 2.06, 95%CI 0.97–4.39, *p* = 0.06; HR 1.58, 95% CI 0.84–2.96, *p* = 0.15, respectively).

**Table 3.** Outcomes of the patients with and without CVD readmission in the first year after dialysis.

Variables	Total (*n* = 1589)	With CVD readmission (*n* = 120)	Without CVD readmission (*n* = 1469)	*p* Value
Follow-up time (month)	47.2 (21.2–82.2)	24.3 (10.8–46.6)	49.0 (22.9–84.4)	**<0.001**
Dropout rate (n, %)	1334 (84%)	114 (95%)	1220 (83%)	**<0.001**
Reasons for dropout (n, %)				**<0.001**
All-cause mortality	500 (31.6%)	75 (62.5%)	425 (29.1%)	**<0.001**
Transfer to hemodialysis	304 (19.1%)	19 (15.8%)	285 (19.4%)	0.40
Transplantation	389 (24.5%)	11 (9.2%)	378 (25.7%)	**<0.001**
Transfer to another center center	71 (4.5%)	5 (4.2%)	66 (4.5%)	0.87
Other reasons	66 (4.2%)	4 (3.3%)	62 (4.2%)	0.81
Reasons for mortality (n, %)	500 (31.6%)	75 (62.5%)	425 (29.1%)	**<0.001**
CVD mortality	233 (14.7%)	48 (40%)	185 (12.6%)	**<0.001**
Infection-associated mortality	104 (6.6%)	11 (9.2%)	93 (6.3%)	0.25
Other causes	91 (5.7%)	10 (8.3%)	81 (5.5%)	0.31
Unknown causes	72 (4.5%)	6 (5.0%)	66 (4.5%)	0.82
Technical failure n (%)	305 (19.2%)	19 (15.8%)	286 (19.5%)	0.34

The results are expressed as the median (25th percentile and 75th percentile) or number (%).

CVD: cardiovascular disease. Bold indicates significance at *p* < 0.05.

**Table 4.** Association between CVD readmission within the first year after dialysis and clinical outcomes.

	All-cause mortality		CVD mortality	
	HR (95%CI)	*p* Value	HR (95%CI)	*p* Value
Model 1	4.18 (3.26–5.35)	**<0.001**	6.30 (4.56–8.70)	**<0.001**
Model 2	2.96 (2.30–3.82)	**<0.001**	4.52 (3.25–6.30)	**<0.001**
Model 3	2.66 (1.91–3.70)	**<0.001**	3.42 (2.20–5.31)	**<0.001**
	Infection-related mortality		Technique failure	
Model 1	3.05 (3.62–5.73)	**0.001**	1.66 (1.04–2.65)	**0.03**
Model 2	2.05 (1.08–3.90)	**0.03**	1.60 (1.00–2.55)	0.05
Model 3	2.06 (0.97–4.39)	0.06	1.58 (0.84–2.96)	0.15

Model 1: univariate.

Model 2: adjusted for age and sex.

Model 3: adjusted for model 2 +DM history + CVD history +  hypertension history + BMI +  hemoglobin + albumin + calcium +  phosphorus + TC + triglyceride + HDLC +  LDLC + sodium + potassium + WCCr + RRF.

CVD: cardiovascular disease; DM: diabetes mellitus; BMI body mass index; TC: total cholesterol; HDLC: high-density lipoprotein cholesterol; LDLC: low-density lipoprotein cholesterol; WCCr: total weekly creatinine clearance; RRF: residual renal function.

Bold indicates significance at *p* < 0.05.

## Discussion

To the best of our knowledge, this study is the first to characterize the risk factors and long-term outcomes of CVD readmissions within the first year after dialysis in PD patients. In this study, a total of 496 patients (31.2%) had at least one episode of readmission within the first year after dialysis initiation, and of these readmissions, 120 were due to CVD. CVD readmission affected 7.6% of all patients and accounted for 24.2% of all readmissions within the first year after dialysis initiation.

CVD is a common complication in dialysis patients that is costly and related to morbidity and mortality [11,19–21]. Moreover, previous studies have demonstrated that CVD is a common cause of readmission in dialysis patients. Indeed, CVD accounted for 40–50% of readmissions in HD patients [9,10,22]. Regarding PD, CVD readmission was responsible for nearly 25% of all readmissions within 120 days after dialysis; additionally, 40.8% of these patients tended to have another CVD readmission within the following 30 days [8]. Similarly, our study found that 31.2% of the patients had at least one episode of readmission within the first year after dialysis initiation, and of these readmissions, 24.2% were CVD readmissions. Thus, the identification of modifiable risk factors of CVD readmissions and providing guidance for prevention strategies are of great clinical significance. However, few studies had explored the related risks of CVD readmission or explained the reasons for the occurrence of CVD readmission in PD patients. Furthermore, studies investigating the prevalence of readmissions in different dialysis modalities have presented conflicting results. Some studies showed that PD patients were at higher risks of all-cause readmission than HD patients [6], but some studies yielded a similar readmission rate [14,22]. However, Ross et al. [23] showed that the readmission rate within the first year was 49.5% in HD patients, which was higher than that in our study, but these authors did not provide the prevalence of CVD readmission.

Kidney diseases have been shown to be a risk factor for CVD readmission in patients within 30 days after acute myocardial infarction (AMI) and other diseases [16,24–26]. In the current study, we found that an advanced age, a history of CVD, and a lower albumin level were independently associated with a higher risk of first-year CVD readmission. Our study is consistent with previous studies showing that patients who have a history of CVD and peripheral arterial disease are more likely to have all-cause and pulmonary edema readmission [8,10,23]. Similarly, Lin et al. [2] demonstrated that the Charlson comorbidity index (CCI) was independently associated with readmissions both in PD and HD and can be a factor used to stratify the risk of readmission. Additionally, in Chan’s study [9], AMI accounted for 21.8% of readmissions, suggesting that a CVD history indicates a greater likelihood of readmission. Furthermore, the associated factors identified in this study investigating CVD readmission within the first year are consistent with the traditional CVD risk factors in patients with kidney disease. For instance, age is independently associated with aortic stiffness and vascular calcification [27,28], which is a strong predictor of CVD. Moreover, a history of CVD reflects endothelial dysfunction [29], inflammation and oxidative stress, which ultimately lead to CVD events [30,31]. In addition, low levels of HDLC are believed to represent an atherogenic lipid profile in the general population and the association between PD and CVD risk has been demonstrated in PD patients [32,33]. Furthermore, serum albumin is known to be associated with arterial stiffness [34], inflammation [35,36], and endothelial dysfunction [37,38]. Altogether, these factors lead to impairment in vasodilation and atherosclerosis, contributing to CVD events [39,40]. Notably patients who initiated dialysis often had increased CVD risks in addition to the already elevated risks from ESRD [11,41]. Dialysis patients in the first year are vulnerable to fluid overload, congestive heart failure and other morbidities [10]. Moreover, as the dialysate solution in PD may result in atrial natriuretic peptide producing and increased formation of advanced glycation end products (AGEs), these would exacerbate congestive heart failure and arterial stiffness in incident PD patients [11]. Nonetheless, poorer RRF and overhydration are more common in dialysis patients than those not on dialysis [11], rendering the population initiating PD more vulnerable to CVD and other events.

Notably, in our study, CVD readmission within the first year had substantially worse all-cause mortality and CVD mortality even after adjusting for several confounders. The patients who had CVD readmission within the first year had a 2.66-fold greater risk of all-cause mortality and 3.42-fold greater risk of CVD mortality than those who did not have readmission. Our findings indicate that incident CAPD patients with CVD readmission in the first year after dialysis initiation had a worse health status than those who did not have readmission; this status is long-lasting and affects long-term outcomes. Interestingly, this finding is consistent with a study investigating HD comparing patients with and without readmission within the first year after dialysis; the patients with readmission had a substantially higher long-term risk of mortality, especially those who had another readmission within the following 30 days [23]. One explanation for our findings could be that patients with readmissions within the first year of dialysis had higher rates of comorbidities and more severe disease conditions, such as inflammation status, oxidative stress, and lipid disorders [23,25,42]. Indeed, the patients in the readmission group in our study were much older, were more likely to have a CVD history, and had lower albumin level. Other potential explanations could be related to the pathological changes in ESRD and additional changes in the aforementioned PD patients. These pathological changes have been demonstrated to be risk factors for all-cause and CVD mortality [26–31]. This study indicates that CVD readmission could be a factor categorizing patients into different risks of long-term outcomes, providing a potential means to identify patients at a higher risk. Additionally, this study suggests that interventions aiming to alleviate the need for readmission may improve outcomes.

This study has some limitations. This study was a single-center retrospective study and involved only PD patients, thus limiting generalizability to other ESRD cohorts and HD patients. Furthermore, although we controlled for many relevant covariates in the analysis, it is possible that some potential confounders, such as socioeconomic status and severity of comorbid conditions, were not well captured in our data. Further studies are needed to identify the modifiable risk factors and long-term outcomes of PD patients with readmission within the first year of dialysis.

## Conclusions

The prevalence of first-year CVD readmission after dialysis initiation in our PD cohort was 7.6%. An advanced age, a CVD history and a lower level of serum albumin were independently associated with first-year CVD readmission. CVD readmission after dialysis initiation was associated with worse all-cause and CVD mortality. However, knowledge regarding how to prevent poor outcomes among PD patients once they experience a readmission is limited. Therefore, identifying modifiable factors associated with readmission in the first year is an important step for developing appropriate interventions for this high-risk population.
