# Correction

**DOI:** 10.1080/0886022X.2021.1890880

**Published:** 2021-02-21

**Article title:** Risk factors and outcomes of cardiovascular disease readmission within the first year after dialysis in peritoneal dialysis patients

**Authors:** Jianbo Li, Naya Huang, Zhong Zhong, Dan Wang, Zhen Ai, Lanping Jiang and Fengxian Huang

**Journal:** *Renal Failure*

**Citation details:** Vol. 43, Number 1, pages 159–167

**DOI:** 10.1080/0886022X.2020.1866009

When the above article was published online, the authors were listed as: Jianbo Li, Naya Huang, Zhong Zhong, Pema Joe, Dan Wang, Zhen Ai, Lisha Wu, Lanping Jiang and Fengxian Huang. However, since publication Pema Joe and Lisha Wu have requested to be removed from the author list because although they helped with the preliminary work related to this study, they do not meet the authorship criteria for the full research represented in this article. This has been agreed with and approved by all the other authors of this article.

The updated author list is now:

Jianbo Li, Naya Huang, Zhong Zhong, Dan Wang, Zhen Ai, Lanping Jiang and Fengxian Huang

The affiliation c has been removed as the remaining authors was not affiliated with Linzhi People’s Hospital.

The online version has been corrected.

